# Supplementary material for: Causal Relationship between Aging and Anorexia Nervosa: A White-Matter-Microstructure-Mediated Mendelian Randomization Analysis
Source: Biomedicines. 2024 Aug 16;12(8):1874. doi: 10.3390/biomedicines12081874 (PMC11351342; doi:10.3390/biomedicines12081874)
Supplement: Supplementary file 1 [file biomedicines-12-01874-s001.zip › Supplementary Figure/Figure S4/Singe SNP plot of MR analysis for TL to ubm-b-1757.pdf]

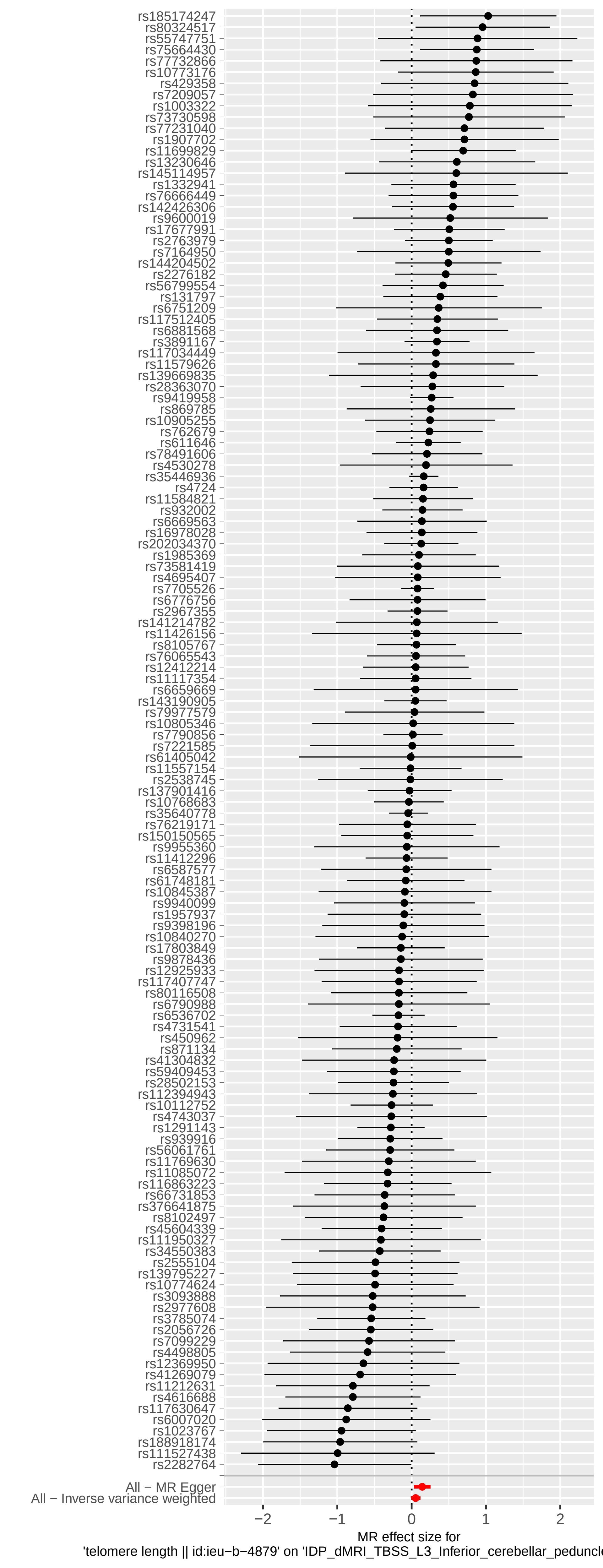
rs185174247
rs80324517
rs55747751
rs75664430
rs77732866
rs10773176
rs429358
rs7209057
rs1003322
rs73730598
rs77231040
rs1907702
rs11699829
rs13230646
rs145114957
rs1332941
rs76666449
rs142426306
rs9600019
rs17677991
rs2763979
rs7164950
rs144204502
rs2276182
rs56799554
rs131797
rs6751209
rs117512405
rs6881568
rs3891167
rs117034449
rs11579626
rs139669835
rs28363070
rs9419958
rs869785
rs10905255
rs762679
rs611646
rs78491606
rs4530278
rs35446936
rs4724
rs11584821
rs932002
rs6669563
rs16978028
rs202034370
rs1985369
rs73581419
rs4695407
rs7705526
rs6776756
rs2967355
rs141214782
rs11426156
rs8105767
rs76065543
rs12412214
rs11117354
rs6659669
rs143190905
rs79977579
rs10805346
rs7790856
rs7221585
rs61405042
rs11557154
rs2538745
rs137901416
rs10768683
rs35640778
rs76219171
rs150150565
rs9955360
rs11412296
rs6587577
rs61748181
rs10845387
rs9940099
rs1957937
rs9398196
rs10840270
rs17803849
rs9878436
rs12925933
rs117407747
rs80116508
rs6790988
rs6536702
rs4731541
rs450962
rs871134
rs41304832
rs59409453
rs28502153
rs112394943
rs10112752
rs4743037
rs1291143
rs939916
rs56061761
rs11769630
rs11085072
rs116863223
rs66731853
rs376641875
rs8102497
rs45604339
rs111950327
rs34550383
rs2555104
rs139795227
rs10774624
rs3093888
rs2977608
rs3785074
rs2056726
rs7099229
rs4498805
rs12369950
rs41269079
rs11212631
rs4616688
rs117630647
rs6007020
rs1023767
rs188918174
rs111527438
rs2282764
All − MR Egger
All − Inverse variance weighted
−2
−1
0
1
2
MR effect size for
'telomere length || id:ieu−b−4879' on 'IDP_dMRI_TBSS_L3_Inferior_cerebellar_peduncl
